# Selected Biological Medicinal Products and Their Veterinary Use

**DOI:** 10.3390/ani10122343

**Published:** 2020-12-09

**Authors:** Aleksandra Zygmuntowicz, Artur Burmańczuk, Włodzimierz Markiewicz

**Affiliations:** 1 Department of Pharmacology and Toxicology, Faculty of Veterinary Medicine, University of Warmia and Mazury in Olsztyn, Oczapowskiego 13, 10-718 Olsztyn, Poland; aleksandra.zygmuntowicz@uwm.edu.pl; 2 Sub-Department of Pharmacology, Toxicology and Environmental Protection, Faculty of Veterinary Medicine, University of Life Sciences in Lublin, 12 Akademicka St., 20-950 Lublin, Poland; artur.burmanczuk@up.lublin.pl

**Keywords:** biological medicinal products, bio-similar drugs, monoclonal antibodies, fusion proteins, active substance

## Abstract

**Simple Summary:**

Biological drugs are a generation of drugs that have developed thanks to advances in genetic engineering and molecular biology. Biological drugs are proteins derived from living cells or obtained through the use of genetic engineering methods with a selective and specific mechanism of action. Currently, these drugs are widely used in the treatment of many human diseases, but an increasing number of drugs from this group are also being used in the treatment of animals, mainly in dermatology, rheumatology and oncology.

**Abstract:**

Definitions of biological medicinal products (BMPs) vary depending on the source. BMPs are manufactured using complex biological/biotechnological processes involving living cell lines, tissues and organisms such as microorganisms, plants, humans and even animals. Advances in modern biotechnological methods and genetic engineering have made it possible to search for new drugs with a targeted effect and simultaneous reduction of adverse effects, which has resulted in BMPs dynamically increasing their share in the pharmaceutical market. Currently, these drugs are widely used in the treatment of many human diseases, but an increasing number of drugs of this group are also being used in the treatment of animals, mainly in dermatology, rheumatology and oncology. This article presents the current state of knowledge in the field of biological medicinal products used in animal therapy.

## 1. Introduction

Biological medicinal products (BMPs) are a group of medicines which are developing rapidly owing to the progress of biotechnological methods. In terms of structure, these are proteins with pharmacological activity, derived from living cells or obtained by genetic engineering methods. The action of BMPs involves mimicking the function of normal animal proteins. Moreover, they act as modulators of immune response, because they mobilize the immune potential of the patient in the fight against the disease [1]. Biological drugs include the following groups: vaccines, blood proteins, toxins, recombinant proteins, monoclonal antibodies, growth hormone, insulins, erythropoietin, interferons, growth factors and interleukins. BMPs are used in the treatment or prevention of cardiological, dermatological, rheumatological and oncological diseases, Turner’s syndrome, diabetes, anemia, oncological and neutropenia [2]. According to the United States Department of Agriculture (USDA), veterinary biological products include the following groups: antibody products, bacterins and bacterial extracts, toxoids, bacterin-toxoids, antitoxins, vaccines, vaccines with bacterins/bacterial extracts/toxoids, diagnostic products and miscellaneous [3]. This review is limited mainly to recombinant proteins, monoclonal antibodies and mesenchymal stem cells (approved by European Medicines Agency (EMA)), and miscellaneous group (approved by the USDA).

## 2. Definition of Biological Medicinal Products

The definition of BMPs has been changing with the progress of knowledge. According to the 1902 definition presented by the Food and Drug Administration (FDA), traditional BMPs include therapeutic vaccines, viruses, serums, blood, blood components, toxins and anti-toxins. According to the current FDA definition, biological medicinal products are substances obtained from living organisms (humans, plants, microorganisms, and even animals) by biotechnological methods and by genetic engineering, applied in therapy of both humans and animals [4]. The USDA defines veterinary biological products as products derived from living organisms and produced during biological processes. They are used to prevent, diagnose or treat animal diseases and function through an immune process [5]. The EMA defines BMPs as medicinal products which contain one or more active substances produced by, or obtained from, a living organism [6]. The first BMP produced through recombinant DNA technology was insulin. This peptide hormone was discovered in 1921 and from the following year, was obtained from porcine and bovine pancreases for therapeutic purposes [7]. It is noteworthy that a one-year therapy of one patient with diabetes requires insulin isolated from 100 porcine pancreases. Without recombinant insulin, 20 billion pigs would have to be kept globally to cover the annual demand for insulin for 200 million patients [8]. The world population of pigs decreased from 766.6 million in the previous year to 677.6 million in January 2020 [9]. Owing to the progress in molecular biology and genetic engineering techniques of the 1970s, researchers could conduct studies aimed at obtaining insulin by culturing genetically modified bacteria, *Escherichia coli*, and yeast, *Saccharomyces cerevisiae* and *Pichia pastoris*, (by incorporating a plasmid with the gene encoding human insulin into the genome) which resulted in introducing the first recombinant drug on the market in 1982 [10,11]. Increasing importance of recombinant medicines is testified to by the fact that in 2019 EMA approved 66 new therapeutic drugs, 18 of which were recombinant drugs, including 11 monoclonal antibodies [12]. In addition, the FDA approved 48 new drugs, 9 of which were recombinant drugs, including 8 monoclonal antibodies [13]. The above drugs have been used to treat people. The only monoclonal antibody used to treat animals is lokivetmab (Zoetis, Louvain-la-Neuve, Belgium), marketed in 2017. Lokivetmab is used in treatment of atopic dermatitis in dogs.

In Europe the marketing authorization applications of BPMs are handled solely through the centralized marketing authorization process, which is co-ordinated by EMA (Regulation (EC) No 726/2004) [14,15]. The assessment of the quality and manufacturing data of BPMs are centered to Biologics Working Party (BWP) at the EMA, whereas the Committee for Human Medicinal Products (CHMP) has the overall responsibility of the marketing authorization applications (MAA) assessment [16]. In the USA, the FDA Veterinary Medicine Center (CVM) oversees the approval and introduction of new animal medicines [17]. However, veterinary biologics, including vaccines for animal diseases, are regulated by the U.S. Department of Agriculture (USDA) Animal and Plant Health Inspection Service (APHIS) [18].

In the production of subsequent biological or biosimilars, non-patented production processes must be used. The drugs received are referred to as “follow-on biologics” or “biosimilars” depending on how they were approved by the FDA. However, they are not referred to as “bioidentical” or “generic” because although they are similar, they are not identical to the reference product, which is the original biological drug, and may show differences in immunogenicity, efficacy and safety. The term “generic” refers to drugs that are identical to the active ingredient of an innovative drug and are the result of chemical reactions, not biological processes. In addition, the FDA developed the Public Health Service Act (PHS Act), which specifies two levels of biosimilar drugs [19]. The first is biosimilar medicine, defined as a biological product that is FDA approved because it is similar to an FDA approved biological reference product and has been shown to have no clinical differences from this product [17]. The first biosimilar drug approved by EMA and placed on the European market in 2006 was the human recombinant growth hormone in the Omnitrope (Sandoz GmbH, Langkampfen, Austria) product, which is a generic drug for Genotropin. The second level, however, is interchangeable biosimilar products, defined as a biosimilar product to an FDA approved reference medicine and it is possible that they have the same clinical effect as the reference medicine and can therefore be used interchangeably [20]. However, no interchangeable biosimilar products have been approved.

## 3. Classification of Biological Medicinal Products

There are several groups of BMPs: monoclonal antibodies, stem cells, fusion (chimeric) proteins, recombinant proteins and antisense oligonucleotides [1,21].

### 3.1. Monoclonal Antibodies

Monoclonal antibodies are immunoglobulins, which specifically bind to proteins located on the surface of cells and contribute to the immune response. They act as immunosuppressants or immunostimulants [22]. The first monoclonal antibodies were of murine origin; however, when introduced to the human body, they produced an immune response against the foreign-species murine immunoglobin (HAMA—human anti-mouse antibody response). For this reason, attempts at humanization of antibodies were made to reduce their immunogenicity [1]. They resulted in obtaining chimeric antibodies, which were in 65–90% human antibodies (the percentages relate to amino acid sequence conservation), by replacing a fixed region of the heavy and light chain of the murine antibody with similar fragments of the human antibody—that is, only the replaced regions are of murine origin. Subsequently, humanized antibodies were produced which were 90–95% human; they were obtained by leaving only the antigen-binding regions in the murine antibody molecule. Currently, fully human antibodies are available, produced by transgenic mice, in which loci of murine DNA were replaced with DNA encoding human immunoglobulins [23]. The names of monoclonal antibodies end with -ab; apart from that, the name ending identifies the antibody type: chimeric antibodies, -ximab; humanized antibodies, -zumab; human antibodies, -umab [1,21].

The monoclonal antibody (mAb) timing system has been developed, resulting in an international non-proprietary name (INN) introduced at the request of the World Health Organization (WHO) or name adoption (USAN) at the request of the American Medical Association (AMA) in the United States [24,25,26]. According to these guidelines, the name mAb consists of the prefix chosen, the target sub-element and the end of the universal core “-mab”. For monoclonal antibodies used in veterinary medicine, the target element is ‘vet’ [27].

### 3.2. Stem Cells

Mesenchymal stem cells (MSCs) are multipotent cells, with capacity for self-renewal and differentiation that derive from the embryonic layer of the mesoderm and under certain conditions they can differentiate into can myocytes, neural cells, osteoblasts, chondrocytes [28]. In animals, two classes of stem cells have been identified: embryonic stem cells (ESC) and adult stem cells (ASC) [29], which include hematopoietic stem cells, mesenchymal stem cells and progenitor cells [30]. In veterinary medicine, MSCs are used for treatment of tendon and ligament injuries, and joint diseases, with significant clinical relevance in horses and for orthopedic applications in dogs. Most often, MSCs are isolated from: adipose tissue, peripheral blood, umbilical cord, muscle, bone marrow, synovium [31]. The low immunogenicity of these cells suggests that MSCs can be transplanted universally without matching between donors and recipients. The transplantation possibilities for peripheral blood MSCs include allogeneic stem cells being injected from another donor animal from the some species, autologous stem cell being injected within the same animal and xenogeneic stem cell being injected from another donor animal from another species [32].

### 3.3. Fusion Proteins

The molecules of fusion proteins consist of two elements which make up a structure resembling an antibody. The first part has two binding domains which recognize a specific receptor protein. The other part is the Fc fragment of human immunoglobulin and it stabilizes the whole structure. The immunogenicity of these proteins is very low [22]. The names of fusion proteins end with -cept [1,33]. However, fusion proteins are not used in veterinary medicine.

### 3.4. Recombinant Proteins

Recombinant proteins are replicas of natural proteins or their fragments. They act specifically through cell receptors, inducing a precise immune response. This group includes interferons, interleukins, growth factors and protein hormones [22]. The names of recombinant proteins start with prefix rhu- [1,34].

### 3.5. Antisense Oligonucleotide

The antisense oligonucleotide group contains few drugs. These are short synthetic molecules, 12–30 nucleotides long, whose mechanism of action involves inhibition of gene expression as a result of blocking the relevant matrix RNA (mRNA) by an external complementary fragment of RNA, which binds selectively with mRNA of the gene of interest, blocks it and, in effect, prevents production of the relevant protein [35]. These drugs are indicated in selected neurodegenerative diseases [36], viral infections [37], cancers [38] and familial hypercholesterolemia [39].

## 4. Biological Medicinal Products in Veterinary Use

### 4.1. BMPs Approved by EMA for Veterinary Use

BMPs approved by EMA (European Medicines Agency) for veterinary use are listed in Table 1.

Virbagen Omega (Virbac, Carros, France) is available as lyophilizate with a solvent, from which suspension for injection is prepared. Effectiveness: a reduction in mortality of 4.4–6.4 times was observed in the treatment of dogs with enteric form of parvovirosis. In the treatment of cats infected with feline leukaemia virus (FeLV), a reduction in clinical symptoms was observed over four months and a reduction in mortality. In cats infected with feline immunodeficiency virus (FIV), mortality was low and did not change as a result of treatment [40] The effectiveness of the preparation in dogs and cats has been confirmed by many authors [41,42,43]. Dogs with parvoviral enteritis given intravenous omega interferon showed a significant improvement in clinical symptoms and a reduction in mortality by 4.4 times compared to control animals [41]. Cats with clinical signs related to feline leukemia virus (FeLV) infection and FeLV co-infection/feline immunodeficiency virus (FIV) receiving subcutaneous recombinant interferon were shown to have significantly lower mortality rates compared to the control group (39% versus 59% in 9 months and 47% compared to 59% in 12 months after therapy) [42]. In dogs receiving Virbagen omega subcutaneously, mean Canine Atopic Dermatitis Extent and Severity Index (CADESI) values improved from 42 on day 0 to 27 on day 120, overall by 36%. Pruritus scores decreased from 5.5 to 4.2, overall by 24%. Mean treatment outcomes slightly increased from 18 to 20. Overall outcomes improved from a mean of 65 to 52, overall by 21% [43].

TruScient, (Zoetis, Louvain-la-Neuve, Belgium) available as lyophilizate and a solvent for the preparation of a solution, was another product placed on the market. Effectiveness: simultaneous treatment of dogs with TruScient together with standard surgical care shortened the time needed for healing fractures. After 18 weeks, the fractures of all the dogs treated with TruScient together with standard surgical care (84) were seen to have healed, compared with 95% of the dogs treated with standard surgical care alone (40 out of 42). The product must not be used in dogs with a known hypersensitivity to the active substance or to any of the excipients or in dogs that are skeletally immature, have an active infection at the operative site, pathological fracture, or any active malignancy [44]. There is no information in the available literature describing the effects of recombinant human bone morphogenetic protein-2 for the treatment of open bone fractures in animals. A human study showed that 1.50 mg/mL of this substance was well above the standard of care in reducing the incidence of secondary interventions and general invasive procedures, accelerating fractures and wound healing, and reducing the rate of infections in patients with open fractures tibia [45]. TruScient was withdrawn from the European Union market at a request of the marketing authorization holder.

Subsequently, ProZinc (Boehringer Ingelheim Vetmedica GmbH, Duluth, GA, USA) was introduced to the market; it is in the form of a suspension for injection. Effectiveness: in a field study involving diabetic cats of different ages and breeds, after 6 weeks of treatment with ProZinc average blood sugar levels decreased and clinical symptoms improved. In an EU field study involving diabetic dogs, ProZinc was as effective as approved veterinary insulin. There has been an improvement in the measurement results of at least one blood sugar indicator and amelioration of at least one of three clinical symptoms: body weight, polyuria and polydipsia. The product must not be used in animals with hypersensitivity to the active substance or in emergencies associated with diabetic ketoacidosis [46]. It has been shown that in diabetic cats treated with protamine-zinc insulin, the probability of remission was 56.2%, and the median survival time (time from diagnosis to death) was 1488 days [47], which confirms the effectiveness of this drug.

Oncept IL-2 (Merial, Lyon, France) was introduced to the market in the same year (2013); it is available as lyophilizate and a solvent for the preparation of a suspension for injection. Effectiveness: the studies showed that in cats treated with Oncept IL-2, tumors returned longer (over 730 days based on mean range value) compared to control cats (287 days). Oncept IL-2 reduced the risk of relapse six months after starting treatment by approximately 56% after a year and 65% after two years [48]. It was shown that the recurrence of idiopathic fibrosarcomas was observed in 28% of cats receiving recombinant human interleukin-2 in the Canarypox virus compared to 61% of control animals during the 12-month follow-up after surgery [49].

Imrestor (Elanco GmbH, Cuxhaven, Germany) is another product available on the market; it is in the form of a solution for injections. Effectiveness: in a field study involving 2465 cows the incidence of clinical mastitis during days 3 to 30 of milk production was 9.1% (113/1235) in the Imrestor group compared with 12.4% (152/1230) in the group receiving indifferent injection. The relative reduction in incidence of mastitis was 26%. The product is contraindicated in cows with hypersensitivity to the active substance [50]. It was observed that animals treated with pegbovigrastim showed a 4- to 5-fold increase in circulating neutrophils within 24 h of starting treatment. Animals receiving pegbovigrastim showed a 35% reduction in the incidence of clinical mastitis compared to the control group during the first 30 days of lactation [51].

Subsequently, the product Cytopoint (Zoetis, Louvain-la-Neuve, Belgium) was introduced to the market, which is available as a solution for injections. Effectiveness: in a field study involving dogs with atopic dermatitis, 142 dogs received Cytopoint every month for 3 months, and 132 dogs were treated with ciclosporin (a drug approved for the treatment of atopic dermatitis). Cytopoint was as effective as ciclosporin in treating itchy skin. After 28 days, the pruritus score decreased by 52% in dogs receiving Cytopoint and 44% in dogs receiving cyclosporin. The product is contraindicated in dogs with hypersensitivity to the active substance and in those with a body weight under 3 kg [52]. A 51.9% reduction in pruritus was demonstrated in dogs treated with lokivetmab compared to 43.72% in dogs treated with cyclosporine after 28 days of dosing [53]. This randomized clinical trial confirms the effectiveness of lokivetmab in dogs with atopic dermatitis.

Arti-Cell Forte (Global Stem cell Technology (GST) NV, Evergem, Belgium) was introduced to the market one year later; it has the form of a suspension for injection. Effectiveness: in a field study involving horses with a history of lameness lasting 2 to 6 months, 50 horses were treated with Arti-Cell Forte and 25 received a placebo (dummy injection) of saline in one fetlock joint. All horses received intravenous ketoprofen, a medicine to relieve pain and inflammation, at the time of treatment. Six weeks after treatment 68% of the horses treated with Arti-Cell Forte had a lameness score reduction by 2 or 3 lameness grades compared to none of the horses in the control group. The product must not be used in animals with hypersensitivity to the active substance [54]. Significant improvement in lameness was observed in horses receiving intra-articular injections with chondrogen-induced mesenchymal stem cells and equine allogeneic plasma compared to the saline control group [55] which confirms the efficacy of this drug in the treatment of osteoarthritis.

The latest product that has obtained the marketing authorization is HorStem (Industrial Ventorro del Cano, Madrid, Spain). It is a suspension for injection. Effectiveness: in a field study of horses with mild to moderate osteoarthritis, 16 horses received an injection of HorStem into the affected joint and 17 horses received a placebo (dummy) injection. Horses were examined at days 14, 35 and 63. Twelve HorStem-treated horses were successfully treated (success rate 75%) at day 63 compared to only four of the control group (success rate 25%). A contraindication to the use of the preparation is hypersensitivity to the active substance [56]. The effectiveness of the equine umbilical cord mesenchymal stem cells was described in previous studies, which demonstrated that 77% of horses receiving these cells returned to work at the same or higher levels [57].

### 4.2. BMPs Approved by USDA for Veterinary Use

According to the USDA there are nine groups of BMPs and the last one (miscellaneous) is characterized in this subsection and in Table 2.

Eqstim (Neogen Corporation, Lexington, KY, USA) is available as a suspension for injection [58]. In a study carried out on horses that received *Propionibacterium acnes*, the immunostimulatory and immunomodulatory properties were characterized by increased expression of CD4 + T cells and lymphokine activated killing (LAK) activity in peripheral blood and bronchoalveolar fluid, increased nonopsonized phagocytosis in peripheral blood leukocytes and decreased pulmonary cellularity [59].

Vetimmune (Sass & Sass, Inc, Oak Ridge, TN, USA) is available as an oral solution [60]. The effectiveness of a polyprenyl immunostimulant was confirmed (in the double-blinded, randomized, placebo-controlled clinical trials in cats experimentally infected with feline herpesvirus type 1 [61].

Oncept (Merial, Lyon, France) is available as a vaccine [62]. The active substance is xenogenic plasmid DNA containing canine DNA of human tyrosinase (huTyr). The efficacy of a vaccine containing plasmid DNA with an insert encoding human tyrosinase confirms data published by Grosenbaugh et al. [63] in which the survival time of dogs suffering from oral malignant melanoma was significantly improved after treatment with the huTyr vaccine.

Zelnate (Diamond Animal Health, Inc., Des Moines, IA, USA) is available as a lyophilisate with two solvents, from which a solution for injection is prepared. The active substance of the preparation is produced by bacterial plasmid DNA with a liposomal carrier [64]. However, the new available data indicate that Zelnate does not appear to be an effective in the metaphylactic therapy of the bovine respiratory disease [65].

Victrio (Bayer AG, Leverkusen, Germany) is available as a lyophilisate with two solvents that are made into a suspension for injection. The active substance of the product is produced by bacterial plasmid DNA with a liposome carrier [66]. It is demonstrated that Victrio specifically and potently activates recombinant chicken toll-like receptor 21 (TLR21) in a nuclear factor kappa B reporter gene assay [67].

Staphage Lysate (Delmond Laboratories, Swarthmore, PA, USA) is available as a vaccine [68]. In dogs with canine atopic dermatitis (CAD) and recurrent bacterial pyoderma that received Staphylococcus aureus Phage Lysate Staphage Lysate an effectiveness of 83.33% for the control of pruritus along with regression of the lesions was observed which confirms the efficiency of such a treatment [69].

Immunocidin Equine (NovaVive Inc., Greater Napanee, ON, Canada) is available as a suspension for injection [70]. A study in horses with sarcoids that received Immunocidin Equine showed that out of 17 cases, nine cases were completely healed (52.9%), three had improved (tumors shrank) (17.6%), and three cases had weight loss, tumors grew or did not resolve (17.6%), and in one case with two tumors, one tumor resolved and the other tumor had a slight regrowth [71].

### 4.3. Hormone Drugs

As this listing shows, the number of BMPs (especially recombinant drugs) approved by EMA and USDA in recent years for animal use is relatively small. This is a consequence of several facts, one of them being that the cost of introducing an original drug to the market is very high; therefore, the target group of patients (animals) should be large enough to ensure a sufficiently large market with a high drug price. Another reason could be the ease of obtaining effective active substances. Hormonal drugs of the gonadotropin group could be a good example. Currently recombinant follitropin alfa (Gonal-F, Merck Serono S.p.A., Modugno, Italy; Luveris, Merck Serono S.p.A., Modugno, Italy) and beta (Follistim, Merck Sharp & Dohme Corp., Whitehouse Station, NJ, USA; and Puregon, N.V. Organon, Oss, Netherlands) obtained from Chinese hamster ovary cells is used in humans, whereas follitropin and lutropin obtained from porcine pituitary extracts are used in animals (Table 3). Moreover, non-pituitary gonadotropins are commonly used in animals; they occur in products containing gonadotropin from a pregnant mare (eCG) or chorionic gonadotropin from pregnant women’s urine (hCG) (Table 3). The above shows that easily available gonadotropins from natural sources are used in animals.

### 4.4. Monoclonal Antibodies

The literature contains information on attempts to use monoclonal antibodies in animal therapy. Antibodies neutralizing beta-hemolysin produced by *Staphylococcus aureus* were used in cattle therapy [76]. Antibodies reducing the virulence of the avian flu virus (H5N1) were used in poultry treatment [77]. Neutralizing monoclonal antibody against flu virus A was used in pig treatment [78]. Antibodies against fibronectin A binding protein and against the clumping factor A were used in cattle treatment [79], which prevented the development of mastitis in cattle. Antibodies against infectious bursal disease virus (IBDV) were used in poultry treatment [80]. However, despite data suggesting the potential for therapeutic use of monoclonal antibodies in the treatment of selected animal diseases, there is still no market for commercially available products, with work on application of this group of drugs currently underway. Two monoclonal antibodies inhibiting the nerve growth factor (NGF) are undergoing clinical trials. These include ranevetmab (NV-01) for dogs and frunevetmab (NV-02) for cats, which are intended for the treatment of chronic pain and degenerative joint disease in cats and dogs [81].

## 5. Conclusions

In conclusion, the development of biotechnological methods provides new therapeutic opportunities; however, the number of drugs used in animal treatment introduced on the market is much smaller compared to drugs for human use. However, data suggest that the market will develop steadily and that new drugs are likely to be introduced in the near future for the treatment of skin diseases, inflammation of the muscular and articular system, cancers and immune diseases. The introduction of recombinant medicinal products, in particular, opens new therapeutic possibilities, which could not be possible with traditional allopathic therapy. This concerns, e.g., viral diseases such as canine parvovirus or feline FeLV and FIV, for which the recombinant interferon omega is the only drug registered in Europe. However, the application of biologic therapies is significantly limited by their high cost in the case of recombinant drugs. Moreover, further studies are necessary concerning the safety of these drugs. Moreover, further studies are necessary concerning the safety of these drugs. This is particularly important in the case of recombinant proteins, which may be characterized by high immunogenicity and could lead to undesirable results, e.g., anaphylactic shock.

➢ Advantages
  - Effectiveness;
  - Safety;
  - New therapeutic possibilities;
  - Reduced time of placing on the market;
  - Potential for reduced costs,
➢ Disadvantages
  - Difficult to guarantee proper structure (folding);
  - Immunogenicity;
  - Digested in the gastrointestinal tract;
  - Limited stability in solutions;
  - The need to apply modern technologies (costs).

## Figures and Tables

**Table 1:** Veterinary biological medicinal products approved for marketing by the European Medicines Agency (EMA).

The Active Substance/Trade Name/Classification	Country and Year of Admission to Trading	Mode of Action	Doses	Target Animals	Indications for Use	Administration	References
Omega Interferon/Virbagen Omega/recombinant proteins	France, 2001	After injection, interferon omega is quickly bound to cells infected by virus that the mechanism of replication is stopped both by destruction of mRNA and by inactivation of translation proteins as a result of activating 2’5’-oligo-adenyl synthase.	5,000,000 IU */vial or 10,000,000 IU/vial	Dogs, cats	Parvovirosis (enteric form) in dogs; cats infected with FeLV * and/or FIV *.	Dogs—intravenous injection, once daily, for three consecutive days, at 2,500,000 IU/kg body weight (b.w.); cats—subcutaneous injection, once daily, for five consecutive days, at 1,000,000 IU/kg b.w., the first 5-day treatment cycle should be followed by two additional 5-day treatment cycles: after 14 days and after 60 days.	[40,41,42,43]
Dibotermin alfa/TruScient/recombinant proteins	Belgium, 2011	Dibotermin alfa (rhBMP-2) is an osteoinductive protein that results in the induction of new bone tissue at the site of implantation. Dibotermin alfa binds to receptors on the surface of mesenchymal cells and causes cells to differentiate into cartilage- and bone-forming cells. The differentiated cells form trabecular bone as the sponge is degraded, with vascular invasion evident at the same time. The bone formation process develops from the outside of the prepared sponge towards the center until the entire prepared sponge is replaced by trabecular bone.	0.66 mg/vial (the solution contained 0.2 mg/mL of dibotermin alfa)	Dogs	Diaphyseal fractures long bones in dogs.	After preparation, the solution is injected to sponges made of type II bovine collagen and grafted directly at the site of a long bone fracture.	[44,45]
Protamine zinc insulin human/ProZinc/recombinant proteins	Germany, 2013	Insulin activates insulin receptors and therewith a complex cell signaling cascade which results in increased glucose uptake into the cells. The main effects of insulin are the reduction in circulating blood glucose concentrations and the storage of fat	40 IU/mL	Cats, dogs	Diabetes mellitus in cats and dogs.	Subcutaneous injection at a recommended dose of 0.2–0.4 IU/kg b.w. every 12 h	[46,47]
Feline interleukin-2 recombinant canarypox virus/Oncept IL-2/recombinant proteins	France, 2013	Oncept IL-2 injected into the tumor bed thus delivers in situ a low dose of feline interleukin-2, which stimulates antitumor immunity while avoiding toxicity associated with systemic treatment.	10^6^/mL	Cats	Fibrosarcoma in cats.	Subcutaneous injection at five places around the tumor removal site: one injection at each corner and one at the center of the square formed in the middle of the post-surgery scar, at approx. 0.2 mL per injection. The treatment regime is based on four injections every week (days 0, 7, 14, and 21), followed by two injections given every two weeks (days 35 and 49). The treatment should start one day earlier before the beginning of radiotherapy	[48,49]
Pegbovigrastim/Imrestor/recombinant proteins	Germany, 2015	The product increases the number of circulating neutrophils. It has also been proved that it enhances myeloperoxidase hydrogen peroxide halide mediated microbiocidal capabilities of neutrophils.	15 IU/vial	Cattle (dairy cows and heifers)	Reduce the risk of clinical mastitis in periparturient dairy cows and heifers.	subcutaneous injection, one pre-filled syringe seven days prior to the expected calving and another 24 h thereafter. Injection of the contents of one pre-filled syringe ensures the administration of pegbovigrastim at 20–40 µg/kg b.w.	[50,51]
Lokivetmab-caninised monoclonal antibody/Cytopoint/monoclonal antibodies	Belgium, 2017	The blocking of IL-31 * by lokivetmab prevents IL-31 from binding to its co-receptor and thereby inhibits IL-31 mediated cell signaling, providing relief from Atopic Dermatitis-related pruritus and anti-inflammatory activity	10 mg/vial, 20 mg/vial, 30 mg/vial or 40 mg/vial	Dogs	Atopic dermatitis in dogs.	Subcutaneous injection, at 1 mg/kg b.w., once a month.	[52,53]
Chondrogenic induced equine allogeneic peripheral blood-derived mesenchymal stem cells/Arti-Cell Forte/stem cells	Belgium, 2018	The chondrogenic induction of the mesenchymal stem cells aims to activate chondroprotective mechanisms, such as the production of extracellular matrix.	1.4–2.5 × 10^6^/mL	Horses	Mild to moderate recurrent lameness associated with non-septic joint inflammation in horses.	Intra-articularly at 2 mL.	[54,55]
Equine umbilical cord mesenchymal stem cells/HorStem/stem cells	Spain, 2019	Mesenchymal stem cells have immunomodulatory and anti-inflammatory properties. They can also affect tissue regeneration	15 × 10^6^/1 mL	Horses	Lameness associated with mild to moderate degenerative joint disease (osteoarthritis) in horses.	By 1 mL intraarticular syringe.	[56,57]

* IU-international unit; FeLV-feline leukaemia virus; FIV-feline immunodeficiency virus; IL-31-Interleukin 31.

**Table 2:** Veterinary biological medicinal products approved by United States Department of Agriculture (USDA) for marketing in the United States.

The Active Substance/Trade Name/Classification	Country and Year of Admission to Trading	Mode of Action	Doses	Target Animals	Indications for Use	Administration	References
Propionibacterium Acnes Immunostimulant/Eqstim/miscellaneous	USA, 2001	Administration of Propionibacterium acnes stimulates macrophage function, natural killer cytotoxicity and cytokine production (IL-1, IFN-gamma *) and provides prophylactic protection against lethal bacterial and viral challenge.	0.4 mg/mL	Horses	Equine Respiratory Disease Complex (ERDC).	Intravenously at 1 mL/100 kg b.w.; the application should be repeated on the third and seventh day, then weekly if needed.	[58,59]
Polyprenyl Immunostimulant/Vetimmune/miscellaneous	USA, 2003	Works by upregulating innate immunity and directing cellular immune response.	2 mg/mL	Cats	Feline herpesvirus infection in cats.	Orally, twice a day, for 15 days in an amount of 0.25 mL/kg b.w.	[60,61]
Canine Melanoma Vaccine, DNA/Oncept/miscellaneous	France, 2010	Upon injection, the plasmid DNA is taken up by muscle cells which then express the human tyrosinase protein. The human tyrosinase protein will stimulate an immune response effective against canine melanoma cells which express tyrosinase.	–	Dogs	Stage II or III canine oral melanoma in dogs.	Transcutaneous vaccination in an amount of 0.4 mL. Initial vaccination is repeated four times, once every two weeks. A booster vaccination is then given every six months.	[62,63]
DNA Immunostimulant/Zelnate/miscellaneous	USA, 2015	Within the cell, the Zelnate liposome breaks down, exposing the DNA and delivering the plasmid to the cytosol. The plasmid DNA PAMP is recognized. This results in activation of the immune cells. The activated immune cell releases cytokines, which help activate other immune cells and tell them to be prepared for the encounter of pathogens	–	Beef cattle, dairy cattle	Bovine respiratory disease due to *Mannheimia haemolytica.*	Intramuscularly in an amount of 2 mL.	[64,65]
DNA Immunostimulant/Victrio/miscellaneous	USA, 2015	Within the cell, the Victrio liposome breaks down, exposing the DNA to endosomal toll-like receptors (TLRs), and delivering the plasmid to the cytosol. The plasmid DNA PAMP is recognized through endosomal TLR21 as well as cytoplasmic DNA recognition pathways. This results in activation of the immune cells. The activated immune cell releases cytokines, which help activate other immune cells and tell them to be prepared for the encounter of pathogens.	–	Chickens	In 18-day-old embryonated eggs as an aid in the reduction of mortality associated with *Escherichia coli* in embryonated eggs and newborn chicks.	Injected into an 18-day-old egg embryo in an amount of 0.05 mL.	[66,67]
*Staphylococcus Aureus* and staphylococcal bacteriophage/Staphage Lysate/miscellaneous	USA, 2016	Enhance the host immune response to *Staphylococcus* spp.	One milliliter of preparation contains: 120–180 million units forming the colony of *S. aureus* and at least 100 million units forming plaques of staphylococcal bacteriophage.	Dogs	Purulent dermatitis in dogs and related staphylococcal hypersensitivity or skin infections caused by microorganisms by the staph component.	Subcutaneous injection: in dogs with allergies, initially 0.2 mL, then an increase of 0.2 mL once a week to 1.0 mL (a total of 5 injections). Once 1.0 mL is reached, weekly 1.0 mL injections are repeated for approximately 10–12 weeks; in non-allergic dogs 0.5 mL 2 times a week for 10 to 12 weeks and then 0.5 to 1.0 mL every 1 or 2 weeks. It is recommended to take antibiotics concomitantly for an initial period of 4 to 6 weeks	[68,69]
Mycobacterium Cell Wall Fraction Immunostimulant/Immunocidin Equine/miscellaneous	USA, 2017	Mycobacterium phlei cell wall fractions exert indirect anticancer activity by stimulating macrophages and lymphocytes leading to the release of anti-tumour cytokines. In addition, it act directly by inducing apoptosis of cancer cells		Horses	Immunotherapy of sarcoid tumors in horses.	1 mL of product per cubic centimeter of tumor; treatment should be repeated every 1 to 3 weeks until the tumor is resolved (generally repeated at 10- to 14-day intervals).	[70,71]

* IFN-gamma—Interferon-gamma.

**Table 3:** Examples of hormone drugs for animal use.

Specimen	The Active Substance	Target Animals	Year of Admission to Trading	Recording Company	Reference
Pluset^®^	Follitropin (FSH) and lutropin (LH); product pig pituitary gland extract	cows, goats, sheep	1990	Laboratorios, Calier, S.A, Barcelona, Spain	[72]
PG600^®^	pregnant mare gonadotropin (eCG) and chorionic Gonadotropin from pregnant women (hCG)	pigs (sows and gilts)	1997	Intervet, International, B.V., Boxmeer, Netherlands	[73]
Folligon^®^	Pregnant mare, serum gonadotropin (eCG)	cattle, sheep, pigs, rabbits	1998	Intervet International B.V., Boxmeer, Netherlands	[74]
Folltropin-V^®^	Follitropin (FSH); pig pituitary gland extract	cows, sexually mature heifers	2000	Bioniche Animal Health; distribution Vétoquinol -A. Inc., Lavaltrie, Canada	[75]

## References

[1] Sobell J.M. (2005). Overview of biologic agents in medicine and dermatology. Semin. Cutan. Med. Surg..

[2] Hansel T.T., Kropshofer H., Singer T. (2010). The safety and side effects of monoclonal antibodies. Nat. Rev. Drug. Discov..

[3] United States Department of Agriculture (USDA) (2020). Veterinary Biological Products. https://www.aphis.usda.gov/animal_health/vet_biologics/publications/currentprodcodebook.pdf.

[4] Public Health Service Act Section 351 (i) (1). https://legcounsel.house.gov/Comps/PHSA-merged.pdf.

[5] United States Department of Agriculture (USDA) Veterinary Biologics. Use and Regulation. https://www.aphis.usda.gov/publications/animal_health/content/printable_version/vet_biologics.pdf.

[6] European Medicines Agency (EMA) EMA/837805/2011. Question and Answers on Biosimilar Medicines (Similar Biological Medicinal Products). https://www.medicinesforeurope.com/wpontent/uploads/2016/03/WC500020062.pdf.

[7] Rosenfeld L. (2002). Insulin: Discovery and controversy. Clin. Chem..

[8] Borkowski L. (2017). Biological biosimilars are available in pharmacies. Apt. Pol..

[9] Statistical Data Number of Pigs Worldwide from 2012 to 2020 (in Million Head). https://www.statista.com/statistics/263964/number-of-pigs-in-selected-countries/.

[10] Baeshen N.A., Baeshen M.N., Sheikh A., Bora R.S., Ahmed M.M.M., Ramadan H.A.I., Saini K.S., Redwan E.M. (2014). Cell factories for insulin production. Microb. Cell. Fact..

[11] Ciarkowska A., Jakubowska A. (2013). *Pichia pastoris* as an expression system for recombinant protein production. Post. Bioch..

[12] European Medicines Agency (EMA) Human Medicines Highlights 2019. https://www.ema.europa.eu/en/documents/report/human-medicines-highlights-2019_en.pdf.

[13] Food and Drug Administration (FDA) New Drug Therapy Approvals 2019. https://www.fda.gov/media/134493/download.

[14] Regulation (EC) No 726/2004 of the European Parliament and of the Council of 31 March 2004 Laying Down Community Procedures for the Authorisation and Supervision of Medicinal Products for Human and Veterinary Use and Establishing a European Medicines Agency (OJ L 136, 30.4.2004, p. 1–33). https://ec.europa.eu/health/sites/health/files/files/eudralex/vol-1/reg_2004_726/reg_2004_726_pl.pdf.

[15] European Medicines Agency (EMA) EMA/716925/2016. The European Regulatory System for Medicines. A Consistent Approach to Medicines Regulation Across the European Union. https://www.ema.europa.eu/en/documents/leaflet/european-regulatory-system-medicines-european-medicines-agency-consistent-approach-medicines_en.pdf.

[16] Finnish Medicines Agency (FIMEA) Biological Medicinal Products. https://www.fimea.fi/web/en/pharmaceutical_safety_and_information/biological-medicinal-products.

[17] U.S. Food and Drug Administration (FDA) Considerations in Demonstrating Interchangeability with a Reference Product. https://www.fda.gov/media/124907/download.

[18] United States Department of Agriculture (USDA) Veterinary Biologics. https://www.aphis.usda.gov/aphis/ourfocus/animalhealth/veterinary-biologics.

[19] U.S. Food and Drug Administration (FDA) Scientific Considerations in Demonstrating Biosimilarity to a Reference Product. https://www.fda.gov/media/82647/download.

[20] U.S. Food and Drug Administration (FDA) Information for Healthcare Professionals (Drugs). https://www.fda.gov/drugs/resources-you-drugs/information-healthcare-professionals-drugs.

[21] Liberek T., Wołyniec W., Renke M., Weber E., Rutkowski B. (2013). Biopharmaceutics in nephrology. Forum Nefrologiczne.

[22] Singri P., West D., Gordon K. (2002). Biologic therapy for psoriasis. The new therapeutic frontier. Arch. Dermatol..

[23] Lohner M.E., Krueger J.G., Gottlieb A. (1999). Clinical trials of a fully human anti-IL-8 antibody for the treatment of psoriasis. Br. J. Dermatol..

[24] Jones T.D., Carter P.J., Plückthun A., Vásquez M., Holgate R.G.E., Hötzel I., Popplewell A.G., Parren P.W.H.I., Enzelberger M., Rademaker H.J. (2016). The INNs and outs of antibody nonproprietary names. MAbs.

[25] Parren P.W.H.I., Carter P.J., Plückthun A. (2017). Changes to International Non-Proprietary Names for antibody therapeutics 2017 and beyond of mice, men and more. MAbs.

[26] World Health Organization (WHO) Selection Process of INNs. https://www.who.int/medicines/services/inn/selection/en/.

[27] Mayrhofer P., Kunert R. (2019). Nomenclature of humanized mAbs: Early concepts, current challenges and future perspectives. Hum. Antib..

[28] Gao F., Chiu S.M., Motan D.A.L., Zhang Z., Chen L., Ji H.L., Tse H.F., Fu Q.L., Lian Q. (2016). Mesenchymal stem cells and immunomodulation: Current status and future prospects. Cell Death Dis..

[29] Gattegno-Ho D., Argyle S.A., Argyle D.J. (2012). Stem cells and veterinary medicine: Tools to understand diseases and enable tissue regeneration and drug discovery. Vet. J..

[30] Brunt K.R., Weisel R.D., Li R.K. (2012). Stem cells and regenerative medicine—Future perspectives. Can. J. Physiol. Pharmacol..

[31] Dias I.E., Pinto P.O., Barros L.C., Viegas C.A., Dias I.R., Carvalho P.P. (2019). Mesenchymal stem cells therapy in companion animals: Useful for immune-mediated diseases?. BMC Vet. Res..

[32] Newman R.E., Yoo D., LeRoux M.A., Danilkovitch-Miagkova A. (2009). Treatment of Inflammatory Diseases with Mesenchymal Stem Cells. Inflamm. Allergy Drug Targets.

[33] Johnston S.L. (2007). Biologic therapies: What and when?. J. Clin. Path..

[34] Paluchowska E., Owczarek W., Jahnz-Różyk K. (2013). Psoriasis biological treatment in Poland. Zdrowie Publiczne Zarządz..

[35] Crooke S.T. (2017). Molecular mechanisms of antisense oligonucleotides. Nucleic Acid Ther..

[36] Bennett C.F., Krainer A.R., Cleveland D.W. (2019). Antisense Oligonucleotide Therapies for Neurodegenerative Diseases. Annu. Rev. Neurosci..

[37] Lenartowicz E., Nogales A., Kierzek E., Kierzek R., Martínez-Sobrido L., Turner D.H. (2016). Antisense Oligonucleotides Targeting Influenza A Segment 8 Genomic RNA Inhibit Viral Replication. Nucleic Acid Ther..

[38] Moreno P.M.D., Pêgo A.P. (2014). Therapeutic antisense oligonucleotides against cancer: Hurdling to the clinic. Front. Chem..

[39] Agarwala A., Jones P., Nambi V. (2015). The role of antisense oligonucleotide therapy in patients with familial hypercholesterolemia: Risks, benefits, and management recommendations. Curr. Atheroscler. Rep..

[40] Virbagen Omega Summary of Product Characteristics. https://www.ema.europa.eu/en/documents/product-information/virbagen-omega-epar-product-information_en.pdf.

[41] De Mari K., Maynard L., Eun H.M., Lebreux B. (2003). Treatment of canine parvoviral enteritis with interferon-omega in a placebo-controlled field trial. Vet. Rec..

[42] De Mari K., Maynard L., Sanguer A., Lebreux B., Eun H.M. (2004). Therapeutic effects of recombinant feline interferon-omega on feline leukemia virus (FeLV)-infected and FeLV/feline immunodeficiency virus (FIV)-coinfected symptomatic cats. J. Vet. Intern. Med..

[43] Litzlbauer P., Weber K., Mueller R.S. (2014). Oral and subcutaneous therapy of canine atopic dermatitis with recombinant feline interferon omega. Cytokine.

[44] TruScient Summary of Product Characteristics. https://www.ema.europa.eu/en/documents/product-information/truscient-epar-product-information_en.pdf.

[45] Govender S., Csimma C., Genant H.K., Valentin-Opran A. (2002). Recombinant Human Bone Morphogenetic Protein-2 for Treatment of Open Tibial Fractures. J. Bone Joint Surg. Am..

[46] ProZinc Summary of Product Characteristics. https://www.ema.europa.eu/en/documents/product-information/prozinc-epar-product-information_en.pdf.

[47] Restine L.M., Norsworthy G.D., Kass P.H. (2019). Loose-control of diabetes mellitus with protamine zinc insulin in cats: 185 cases (2005–2015). Can. Vet. J..

[48] Oncept IL-2 Summary of Product Characteristics. https://www.ema.europa.eu/en/documents/product-information/oncept-il-2-epar-product-information_en.pdf.

[49] Jourdier T.M., Moste C., Bonnet M.C., Delisle F., Tafani J.P., Devauchelle P., Tartaglia J., Moingeon P. (2003). Local immunotherapy of spontaneous feline fibrosarcomas using recombinant poxviruses expressing interleukin 2 (IL2). Gene Ther..

[50] Imrestor Summary of Product Characteristics. https://www.ema.europa.eu/en/documents/product-information/imrestor-epar-product-information_en.pdf.

[51] Canning P., Hassfurther R., TerHune T., Rogers K., Abbott S., Kolb D. (2017). Efficacy and clinical safety of pegbovigrastim for preventing naturally occurring clinical mastitis in periparturient primiparous and multiparous cows on US commercial dairies. J. Dairy Sci..

[52] Cytopoint Summary of Product Characteristics. https://www.ema.europa.eu/en/documents/product-information/cytopoint-epar-product-information_en.pdf.

[53] Moyaert H., van Brussel L., Borowski S., Escalada M., Mahabir S.P., Walters R.R., Stegemann M.R. (2017). A blinded, randomized clinical trial evaluating the efficacy and safety of lokivetmab compared to ciclosporin in client-owned dogs with atopic dermatitis. Vet. Dermatol..

[54] Arti-Cell Forte Summary of Product Characteristics. https://www.ema.europa.eu/en/documents/product-information/arti-cell-forte-epar-product-information_en.pdf.

[55] Broeckx S.Y., Martens A.M., Bertone A.L., van Brantegem L., Duchateau L., van Hecke L., Dumoulin M., Oosterlinck M., Chiers K., Hussein H. (2019). The use of equine chondrogenic-induced mesenchymal stem cells as a treatment for osteoarthritis: A randomised, double-blinded, placebo-controlled proof-of-concept study. Equine Vet. J..

[56] HorStem Summary of Product Characteristics. https://www.ema.europa.eu/en/documents/product-information/horstem-epar-product-information_en.pdf.

[57] Van Loon V.J.F., Scheffer C.J.W., Genn H.J., Hoogendoorn A.C., Greve J.W. (2014). Clinical follow-up of horses treated with allogeneic equine mesenchymal stem cells derived from umbilical cord blood for different tendon and ligament disorders. Vet. Q..

[58] Eqstim Information Leaflet. https://animalsafety.neogen.com/pdf/prodinfo/eqstim.pdf.

[59] Flaminio M.J.B.F., Rush B.R., Shuman W. (1998). Immunologic function in horses after non-specific immunostimulant administration. Vet. Immunol. Immunopathol..

[60] Vetimmune Information Leaflet. https://vetimmune.com/pi-product-information/.

[61] Legendre A.M., Kuritz T., Heidel R.E., Baylor V.M. (2017). Polyprenyl immunostimulant in feline rhinotracheitis: Randomized placebo-controlled experimental and field safety studies. Front. Vet. Sci..

[62] Oncept Information Leaflet. https://vcahospitals.com/san-francisco/-/media/vca/documents/services/oncology/oncept_tech_detailer_merial_101012.pdf?la=en&hash=CAEF2F9E230862A1BEFB5A1F77F9E2D3.

[63] Grosenbaugh D.A., Leard A.T., Bergman P.J., Klein M.K., Meleo K., Susaneck S., Hess P.R., Jankowski M.K., Jones P.D., Leibman N.F. (2011). Safety and efficacy of a xenogeneic DNA vaccine encoding for human tyrosinase as adjunctive treatment for oral malignant melanoma in dogs following surgical excision of the primary tumor. Am. J. Vet. Res..

[64] Zelnate Information Leaflet. https://www.zelnate.com/static/documents/Zelnate-ChallengeStudy_Detailer.pdf.

[65] Martin B. (2020). Effect of Zelnate Administered as a Metaphylactic upon Initial Processing of High-Risk, Newly Received Beef Calves on Performance and Morbidity. Animal Science Undergraduate Honors Theses. https://scholarworks.uark.edu/anscuht/41.

[66] Victrio Information Leaflet. https://www.bayerlivestock.com/static/documents/Livestock/pdf%20documents/VCT151070_Victrio_Master_Detailer.pdf.

[67] Ilg T. (2020). The immunostimulator Victrio activates chicken toll-like receptor 21. Vet. Immunol. Immunopathol..

[68] Staphage lysaten Information Leaflet. https://delmontlabs.com/wp-content/uploads/2016/11/Package-Insert.pdf.

[69] Solomon S.E.B., de Farias M.R., Pimpao C.T. (2016). Use of *Staphylococcus aureus* Phage Lysate Staphage Lysate (SPL)^®^ for the Control of Recurrent Pyoderma Eczema in Dogs with Atopic Dermatitis. Acta Sci. Vet..

[70] Immunocidin Equine Information Leaflet. http://novavive.ca/uploads/products/files/3923-us-immunocidinequine-brochure-06-17-hrz.pdf.

[71] Caston S.S., Sponseller B.A., Dembek K.A., Hostetter J.M. (2020). Evaluation of Locally Injected *Mycobacterium* Cell Wall Fraction in Horses with Sarcoids. J. Equine Vet. Sci..

[72] Pluset Characteristics of the Veterinary Medicinal Product (ChPLW). http://plw.urpl.gov.pl/files/PLUSET_charakterystyka.pdf.

[73] PG600 Summary of Product Characteristics. https://www.hpra.ie/img/uploaded/swedocuments/LicenseSPC_10996-025-001_17102016100943.pdf.

[74] Folligon Summary of Product Characteristics. https://www.hpra.ie/img/uploaded/swedocuments/LicenseSPC_10996-055-001_21032017164937.pdf.

[75] Folltropin Information Leaflet. http://plw.urpl.gov.pl/files/Folltropin_ulotka_inf.pdf.

[76] Jangra P., Singh A. (2010). *Staphylococcus aureus* β-hemolysin-neutralizing single domain antibody isolated from phage display library of Indian desert camel. Asian Pac. J. Trop. Med..

[77] Zhang T., Wang C.Y., Zhang W., Gao Y.W., Yang S.T., Wang T.C., Zhang R., Qin C., Xia X.Z. (2010). Generation and characterization of a fusion protein of single chain fragment variable antibody against hemagglutinin antigen of avian influenza virus and truncated protamine. Vaccine.

[78] Corti D., Voss J., Gamblin S.J., Codoni G., Macagno A., Jarrossay D., Vachieri S.G., Pinna D., Minola A., Vanzetta F. (2011). A neutralizing antibody selected from plasma cells that binds to group 1 and group 2 influenza A hemagglutinins. Science.

[79] Wang M., Zhang Y., Li B., Zhu J. (2015). Construction of scFv that bind both fibronectin-binding protein A and clumping factor A of *Stapylococcus aureus*. Res. Vet. Sci..

[80] Zhang Y., Yin J., Li T., Zhou B., Xu P., Che R., Liu Y., Cao H., Ye X., Yang Y. (2017). A recombinant avian antibody against VP2 of infectious bursal disease virus protects chicken from viral infection. Res. Vet. Sci..

[81] Enomoto M., Mantyh P.W., Murrell J., Innes J.F., Lascelles B.D.X. (2019). Anti-nerve growth factor monoclonal antibodies for the control of pain in dogs and cats. Vet. Rec..

